# Caesarean section following induction of labour in uncomplicated first births- a population-based cross-sectional analysis of 42,950 births

**DOI:** 10.1186/s12884-016-0869-0

**Published:** 2016-04-27

**Authors:** Mary-Ann Davey, James King

**Affiliations:** Department of Obstetrics and Gynaecology, Monash University, 246 Clayton Rd, Clayton, 3186 Australia; Department of Obstetrics and Gynaecology, University of Melbourne, Parkville, 3052 Australia; Judith Lumley Centre, La Trobe University, 215 Franklin St, Melbourne, 3000 Australia

**Keywords:** Caesarean, Induction, Elective, Primipara, Augmentation, Low risk

## Abstract

**Background:**

The impact of elective induction of labour at term on the increasing caesarean section (CS) rate is unclear. A Cochrane Systematic Review that concluded that elective induction was associated with a reduction in CS was based on trials that mostly reflect outdated obstetric care, or were flawed.

The findings of other studies vary widely in the magnitude and direction of the relationship between elective induction and CS. This inconsistency may be due to the heterogeneity in the methods used to induce or augment labour, such that the relationship with CS is not constant across methods.

**Methods:**

Using validated, routinely-collected data on all births in Victoria in 2000–2005, all singleton, cephalic, first births following uncomplicated pregnancies at 37–40 completed weeks’ gestation (‘standard primiparae’) were identified (*n* = 42,950). As well as comparing induced with non-induced labour, method of birth was compared between those women experiencing spontaneous labour without augmentation, and women undergoing each method of augmentation or induction using adjusted multinomial logistic regression. Proportions, chi-square tests, adjusted Relative Risk Ratios (aRRR) and 95 % confidence intervals are presented.

**Results:**

Ten percent of “standard primiparae” had labour induced for no apparent medical indication. Women whose labour was induced were significantly more likely than those who laboured spontaneously to have a CS (26.5 and 12.5 % respectively (OR 2.54, 95 % CI 2.4, 2.7, *p* < 0.001). After adjustment for maternal age, epidural analgesia, birthweight, gestation, and public/private admission status, each method of induction or augmentation remained associated with a significant increase in the risk of CS (adjusted ORs range 1.48 to 4.13, *p*-values all <0.0001). Perinatal death did not differ by onset of labour.

**Conclusion:**

Induction of labour in medically uncomplicated nulliparous women at term carries a more than doubling of risk of emergency CS, compared with spontaneous labour, with no impact on perinatal mortality. All methods of induction and augmentation of labour were associated with an increase in the rate of CS. Women included in this study had no apparent medical indication for induction of labour or any complication of pregnancy, so the increase in CS was not due to identifiable underlying risk factors. These results suggest that, in the absence of direction from well-designed, contemporary RCTs, minimising unindicated inductions before 41 weeks’ gestation has the potential to reduce the rate of CS.

## Background

The high caesarean section (CS) rate in developed countries is causing widespread concern. In 2011, 32.3 % of women giving birth in Australia did so by CS (33.2 % of first births) [1]. The CS rate in the state of Victoria doubled from 15.3 % in 1985 to 30.6 % in 2006 [1], and was 32.0 % in 2011 [2]. Although CS can be life-saving for the mother and/or baby when used judiciously, it also carries risks for: higher rates of maternal morbidity, mortality and delayed recovery from the birth [3, 4]; difficulty establishing breastfeeding [5]; neonatal morbidity and admission to nursery care [4]; serious placental complications in subsequent pregnancies [6, 7]; and increased costs for the health system [8].

Coinciding with the increase in CS was an increase in the proportion of labours that were induced, from 20 % in Victoria in 1992 to 28 % in 2002 [9] and it has since stabilised at around 25 % [2]. Other countries share this trend. Zhang found a doubling of the induction rate in the United States between 1990 and 1998 [10] though this also appears to have plateaued [11].

Concerned clinicians and policy-makers are keen to identify evidence-based, appropriate strategies to safely reduce the CS rate. Vaginal birth after CS is relatively uncommon with 84.1 % of women in Australia who had a prior CS doing so again in the next birth in 2011 [12] along with 86.5 % of women giving birth in Victorian public hospitals in 2011 whose only prior birth was a CS [13], so strategies that would safely prevent a first CS show great promise for reducing the overall CS rate in the longer term.

The literature on the association between induction of labour and CS is divided. Some observational studies found no increase in the rate of CS following induction of labour [14, 15] while others have found an increase [16–21].

The Cochrane systematic review on this topic [22] was updated in 2012 with a total of 22 randomised controlled trials (RCTs), most of which were conducted in an era before RCT methods were developed to current standards and so have fundamental flaws. The authors reported a reduction in perinatal death with a policy of induction of labour and a lower rate of CS (RR 0.89) and no difference in the rate of admission to SCN/NICU. Other systematic reviews of studies that included indicated and non-indicated inductions of labour found a similar reduction in CS overall in those randomised to induction [23–25]. However the trials in the Mishanina review that looked separately at first births and subsequent births found no difference in CS between those randomised to induction or expectant management [23]. Saccone conducted a review of RCTs of induction or expectant management in uncomplicated multiparous and primiparous women and found no difference in the rate of CS [24].

During the period of this study, three techniques were commonly used individually or in combination to induce labour in Victoria: application of prostaglandins to the cervix if unfavourable, artificial rupture of the fetal membranes (amniotomy), and continuous intravenous infusion of synthetic oxytocin [1]. As well as being used to induce labour, oxytocin infusion and amniotomy were also used to augment labour that had begun spontaneously. Even though some of the same agents are used to induce or augment labour, it is plausible that their effect might differ when used at these different times depending on whether known and unknown pre-labour changes have occurred in the woman’s body.

The overall aim in this study is to describe and analyse the events that follow induction of labour in uncomplicated nulliparous pregnancies. This information would be expected to improve the knowledge of identified risks and benefits, so that pregnant women expecting their first baby at term and their caregivers will be better informed as they consider whether to induce or await spontaneous labour. This paper examines the risk of CS in this low risk group following the induction of labour overall, and following the various individual methods of induction or augmentation of labour in uncomplicated pregnancies at term.

## Methods

Maternal and perinatal data were collected by the midwife attending every birth in the state of Victoria, Australia, of at least 20 weeks’ gestation, or, if gestation was unknown, of at least 400 g birth weight. These were reported using a standard perinatal statistics form to the Victorian Perinatal Data Collection (VPDC), which collects this information on behalf of the Consultative Council on Obstetric and Paediatric Mortality and Morbidity (CCOPMM).

The perinatal form contained information on maternal age, parity, gestation, presentation, plurality, birthweight, sex of the baby, pre-existing maternal medical conditions, complications of pregnancy, onset of labour, indication for induction, agents or techniques used to induce or augment labour, analgesia used, method of birth, public or private admission status, as well as many other variables not used in this analysis.

The accuracy of the VPDC items used in these analyses was previously validated on a random sample of cases in the 2003 data set [26].

Use of the data for the calendar years 2000–2005 inclusive for this project was approved by the Consultative Council on Obstetric and Paediatric Mortality and Morbidity, and the project was approved by the Faculty of Health Sciences, Ethics in Human Research Committee, La Trobe University (approval number FHEC03/146).

In order to compare women with similar levels of risk and no medical indication for induction, according to whether they had labour induced or augmented or a spontaneous onset of labour, cases that did not meet the definition of the “standard primipara” were excluded. For the purpose of this study, a “standard primipara” was defined as being between 20 and 44 years of age, having no complications of pregnancy and no pre-existing diabetes, hypertension, heart disease or mental illness, giving birth for the first time, to a singleton, cephalic-presenting infant with a birthweight between the 10th and 90th centile for sex and gestation, at 37–40 weeks’ gestation. The standard local definition of standard primipara excludes women aged 35 years or older, but given the increasing maternal age at first birth, we extended the definition to include women aged 35–44 years who otherwise met the criteria for the standard primipara. The upper gestational limit was set at 286 days (40 weeks and 6 days) because induction for post-dates at 41 weeks is standard practice in many settings. In order to clarify that we have made these modifications to the local standard primipara definition, we have enclosed the words “standard primipara” in quotes. All births to “standard primiparae” in Victoria from January 2000-December 2005 were included in the study.

Age, parity, plurality, presentation, birthweight and gestation were reported on the perinatal form and entered verbatim into the dataset. The specified maternal medical conditions, as well as pre-eclampsia, gestational diabetes mellitus, pre-labour rupture of the membranes, placental abruption and ‘other APH’ (antepartum haemorrhage) were reported by midwives by ticking a box on the form. The ICD10 code for each of these conditions appeared on the form and was entered into the dataset. Other complications of pregnancy, and indication for induction of labour, were reported as free text in response to the fields ‘other obstetric complications’ and ‘specify indication for induction’ on the form. This text was coded by a health information manager according to ICD10, and the code entered. Up to ten complications of pregnancy could be included. One indication for induction was specified. Forms submitted with missing data, including those with no specified indication for an induced labour, were queried with the hospital, and the data obtained and entered. Women with an entry in any of the ten ‘obstetric complications’ fields were excluded from the “standard primipara” set. Women were also excluded if the indication for induction was an obstetric complication or one of the specified maternal medical conditions.

When labour was induced (e.g. with amniotomy), then an oxytocin infusion commenced some time later, this labour was defined as induced, with the later oxytocin considered to be part of the induction process. When labour began spontaneously, and an oxytocin infusion or amniotomy (or both) were performed some time later, this was considered an augmentation of labour. If the membranes ruptured spontaneously and labour did not follow, an oxytocin infusion administered to stimulate labour was considered by VPDC to be an induction of labour, because there had been no spontaneous onset of labour.

Planned caesarean sections were those that had been arranged before the onset of labour (even if they needed to be moved forward when labour began unexpectedly). Unplanned caesareans were those that took place in response to an emergent problem, whether before labour (e.g. cord prolapse) or during labour.

The small number of women with failed induction of labour were classified as unplanned CS before the onset of labour.

Perinatal mortality included babies who were stillborn and those who died within 28 days of birth. Babies with congenital anomalies are not excluded unless the anomaly was reported as a complication of pregnancy, or as the indication for induction of labour.

In order to explore the effect of each method of induction or augmentation separately in multivariate analysis, a new variable was created with mutually exclusive and exhaustive categories for each combination of onset of labour and method of induction or augmentation. These categories were:

- spontaneous onset, no augmentation;
- spontaneous onset, augmented with amniotomy;
- spontaneous onset, augmented with oxytocin infusion;
- spontaneous onset, augmented with amniotomy and oxytocin infusion;
- labour induced with prostaglandin gel only;
- labour induced with amniotomy only;
- labour induced with oxytocin infusion only;
- labour induced with prostaglandin and amniotomy;
- labour induced with prostaglandin and oxytocin infusion;
- labour induced with amniotomy and oxytocin infusion;
- labour induced with prostaglandin, amniotomy and oxytocin infusion.

Those who had a CS without labour were excluded from multivariate analysis. Descriptive statistics were used to describe the characteristics of the women included in the study. Proportions were compared using Pearson Chi-square and Fisher’s exact tests.

The association between each of these induction/augmentation methods or combination of methods and method of birth was analysed via multinomial logistic regression, adjusting for potential confounders selected a priori. Adjusted relative risk ratios and their 95 % confidence intervals are reported. Maternal age and birthweight were linearly and positively related to CS, so they were included in the model as continuous variables. Gestation, public/private admission status and use of epidural analgesia were included as categorical variables. The very small number of cases with missing data were excluded from multivariate analysis.

Multinomial logistic regression was used because there are three possible outcomes for method of birth – unassisted vaginal birth, operative vaginal birth or CS. This paper presents the results for caesarean compared with unassisted vaginal birth. The Relative Risk Ratio is the output of multinomial logistic regression but is mathematically equivalent to an Odds Ratio.

## Results

**Fig. 1:**
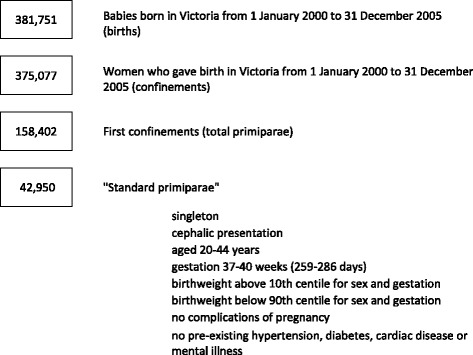
Flow chart in selection of “Standard primiparae”

There were a total of 381,751 births to 375,077 women in Victoria in 2000–2005 of whom 158,402 were first births, and 42,950 met all “standard primipara” inclusion criteria (Figure 1). Fewer than half had a spontaneous labour without augmentation and one in ten had labour induced (Table 1).

**Table 1:** Maternal characteristics and birth details of participants

	n	%
Total	42,950	
Maternal age		
20–24 years	7781	18.1
25–29 years	15,290	35.6
30–34 years	14,637	34.1
35–39 years	4534	10.6
40–44 years	708	1.6
Gestation (completed weeks)		
37	1941	4.5
38	6085	14.2
39	12,186	28.4
40	22,738	52.9
Admission status		
Public	24,110	56.1
Private	18,840	43.9
Type of labour		
Spontaneous with no augmentation	20,117	46.8
Spontaneous and augmented	16,492	38.4
Induced	4182	9.7
No labour	2159	5.0
Type of birth		
Unassisted vaginal	23,407	54.5
Vacuum	5727	13.3
Forceps	5981	13.9
Planned no-labour caesarean	1984	4.6
Planned in-labour caesarean	93	0.2
Unplanned no-labour caesarean	275	0.6
Unplanned in-labour caesarean	5482	12.8
Unknown	1	0
Epidural analgesia in labour (of those who experienced labour, *n* = 40,791)		
No	26,752	65.6
Yes	14,039	34.4

Increasing maternal age and admission as a private patient were associated with induction of labour and pre-labour CS (Table 2).

**Table 2:** Onset of labour by maternal characteristics

	Spontaneous and not augmented		Spontaneous and augmented		Induced		No labour		Total	
	n	%	n	%	n	%	n	%		*p*-value
Maternal age										<0.001
20–24 years	4129	*53.1*	2912	*37.4*	580	*7.5*	160	*2.1*	7781	
25–29 years	7521	*49.2*	5864	*38.4*	1402	*9.2*	503	*3.3*	15,290	
30–34 years	6482	*44.3*	5778	*39.5*	1564	*10.7*	813	*5.6*	14,637	
35–39 years	1751	*38.6*	1756	*38.7*	534	*11.8*	493	*10.9*	4534	
40–44 years	234	*33.1*	182	*25.7*	102	*14.4*	190	*26.8*	708	
*Gestation (completed weeks)*										<0.001
37	1213	*62.5*	520	*26.8*	68	*3.5*	140	*7.2*	1941	
38	2986	*49.1*	1893	*31.1*	299	*4.9*	907	*14.9*	6085	
39	6171	*50.6*	4703	*38.6*	613	*5.0*	699	*5.7*	12,186	
40	9747	*42.9*	9376	*41.2*	3202	*14.1*	413	*1.8*	22,738	
Admission status										<0.001
Public	12,975	*53.8*	9054	*37.6*	1484	*6.2*	597	*2.5*	24,110	
Private	7142	*37.9*	7438	*39.5*	2698	*14.3*	1562	*8.3*	18,840	

Overall, 18.2 % of all “standard primiparae” gave birth by CS (*n* = 7834); and 13.9 % of “standard primiparae” who experienced labour gave birth by CS (*n* = 5675).

Women whose labour was induced were significantly more likely to have a CS than those whose labour was entirely spontaneous (26.5 and 12.5 % respectively) (unadjusted Odds Ratio 2.54, 95 % CI 2.4, 2.7, *p* < 0.0001) (Table 3). Those with a spontaneous onset of labour that was later augmented were significantly more likely to have a CS than those who did not have labour augmented (18.5 and 7.5 % respectively) (Table 4).

**Table 3:** Caesarean section in “standard primiparae” by type of labour

	Caesarean section			Odds ratio	95 % CI	
	n	%	Total			p
Overall	7834	18.2	42,950			
Women who experienced labour	5675	13.9	40,791			
Type of labour						<0.001
Spontaneous (all)	4565	*12.5*	36,609	1.00	ref	
*(not augmented)*	*(1507)*	*(7.5)*	*(20,117)*			
*(augmented)*	*(3058)*	*(18.5)*	*(16,492)*			
Induced	1110	*26.5*	4182	2.54	(2.4, 2.7)

**Table 4:** Relative risk ratios for caesarean section in “standard primiparae” who experienced labour

	RRR	95 % CI	aRRR^b^	95 % CI	*p*-value
Type of labour					
Spontaneous	1	ref gp^a^	1	ref gp^a^	
Aug amniotomy	1.76	(1.6, 1.9)	1.48	(1.3, 1.6)	<0.0001
Aug oxytocin	6.17	(5.7, 6.7)	2.89	(2.6, 3.2)	<0.0001
Aug amniotomy & oxytocin	6.51	(5.9, 7.2)	2.55	(2.3, 2.8)	<0.0001
IOL PG	5.84	(5.0, 6.9)	3.86	(3.2, 4.6)	<0.0001
IOL amniotomy	3.08	(1.8, 5.2)	2.73	(1.6, 4.7)	<0.0001
IOL oxytocin	7.37	(4.4, 12.2)	4.13	(2.4, 7.0)	<0.0001
IOL PG & amniotomy	5.05	(3.9, 6.5)	2.78	(2.1, 3.6)	<0.0001
IOL PG & oxytocin	11.83	(8.4, 16.6)	4.06	(2.8, 5.8)	<0.0001
IOL amniotomy & oxytocin	3.86	(3.3, 4.6)	1.82	(1.5, 2.2)	<0.0001
IOL PG&amniotomy&oxytocin	10.19	(8.8, 11.9)	3.79	(3.2, 4.5)	<0.0001
Mother’s age in years					
per year older	1.132	(1.12, 1.14)	1.088	(1.08, 1.10)	<0.0001
Epidural analgesia in labour					
No	1	ref gp^a^	1	ref gp^a^	
Yes	4.47	(4.2, 4.7)	5.68	(5.3, 6.1)	<0.0001
Birthweight					
per 100 g increase	1.086	(1.08, 1.1)	1.101	(1.09, 1.11)	<0.0001
Gestation (completed weeks)					
37	0.72	(0.6, 0.8)	0.94	(0.8, 1.1)	0.523
38	1.31	(1.2, 1.4)	0.85	(0.8, 0.95)	0.005
39	0.87	(0.8, 0.9)	0.83	(0.8, 0.9)	<0.0001
40	1	ref gp^a^	1	ref gp^a^	
Admission status of mother					
Public	1	ref gp^a^	1	ref gp^a^	
Private	2.47	(2.3, 2.6)	1.11	(1.03, 1.2)	0.003

*Aug* augmented, *IOL* induction of labour, *PG* prostaglandins

^a^ ref gp = reference group

^b^ Relative Risk Ratios after adjustment for all other variables in the table

At a bivariate level each individual method of induction or augmentation of labour was associated with an increased odds of CS compared with spontaneous labour (Table 4). The greatest increase was seen in labours that were induced with both prostaglandin and oxytocin, with or without amniotomy.

CS was also positively associated with a number of other factors: higher maternal age, admission as a private patient, higher birthweight, use of epidural analgesia for pain relief in labour, and use of oxytocin infusions to induce or augment labour (Table 4). The relationship between gestation and CS differed in that babies born at 38 weeks (266 to 272 days) were more likely than any other gestation to be born by CS. The majority of CS performed at 38 weeks were planned procedures (14.3 % of all births in this group at 38 weeks, compared with 9.3 % unplanned). These variables are likely to influence each other in complex ways. For example, women who had labour induced or augmented were three times as likely to use epidural analgesia as those in spontaneous labour (52.2 and 52.4 % for those with induced and augmented labour respectively compared with 18.0 % of those whose labour was neither induced nor augmented –data not shown).

Adjusting for all of the other variables in the model reduced the magnitude of the risk but each method of induction and augmentation of labour remained significantly associated with CS. Women who had labour induced using prostaglandin or oxytocin, alone or in combination with each other without amniotomy, or with a combination of prostaglandin gel, oxytocin infusion and amniotomy, had a more than three-fold increase in the adjusted Relative Risk Ratio of a CS compared with those in spontaneous labour. Augmentation with oxytocin was associated with a more than doubling of the risk of CS, while a ‘simple’ amniotomy augmentation was accompanied by a 48 % increase in risk, though this was probably not a causal relationship (Table 4).

After adjustment there remained strong evidence that use of epidural analgesia in labour was associated with a large increase in CS, and that higher birthweight, older maternal age and admission as a private patient were also associated with an increased risk of caesarean.

Giving birth at 38 and 39 weeks (266 to 279 days) gestation was associated with a significant but slight reduction in the risk of CS, while birth at 37 weeks (259 to 265 days) did not differ from 40 weeks (280 to 286 days) in terms of the risk of CS.

The relationship between onset of labour and CS was graphed separately for those who used, and did not use, epidural analgesia, for public and private patients, and for each week of gestation (not shown). Inspection revealed no interactions.

Infant outcomes will be reported separately, but for “standard primiparae”, perinatal mortality for labour that was induced did not differ from that for spontaneous or augmented labour or pre-labour CS (0.24 per thousand, 0.80 per thousand, 0.97 per thousand and 0.46 per thousand respectively, Fisher’s exact test *p* = 0.537) though the study was not powered to detect a difference in this outcome.

## Discussion

This paper examines the association between the various methods of induction and augmentation of labour and CS in nulliparous women free of existing medical complications (“standard primiparae”) in Victoria, Australia, between January 2000 and December 2005. These women had no apparent medical reason for induction, so could reasonably have had induction withheld or delayed. This analysis provides strong evidence for an increase in CS associated with all methods of elective induction or augmentation of labour. The increase persisted after adjustment for maternal age, use of epidural analgesia in labour, birthweight, gestation and admission as a public or private patient. This information may assist decision-making for women considering induction of labour in the absence of medical indications, as it balances the perceived benefits of induction against the increased risk of CS.

Importantly, perinatal mortality was very rare in this low-risk group and there were no differences between spontaneous, augmented, induced onset of labour or pre-labour CS. Given that we excluded women with identifiable risk factors for stillbirth (those with any complication of pregnancy, those with fetal growth restriction and those who had reached 41 weeks’ gestation), we think it unlikely that the primary aim of these inductions of labour would have been prevention of perinatal mortality. The sample size required to demonstrate a difference in perinatal mortality would have been very large, and it was not the primary aim of the analysis. In light of the small number of deaths in this low risk cohort, it would not be justified to draw conclusions about apparent differences between groups.

The findings are generalisable in contemporary practice with similar approaches to induction and CS. Importantly, the rate of induction of labour has not increased in Australia since these data were collected, and the CS rate has changed little, indicating that the threshold for deciding on CS has not altered appreciably. The results are not generalisable to women who have a medical indication for induction of labour as they were excluded from this analysis.

These findings are at odds with a number of systematic reviews of RCTs of induction of labour and CS [22–25, 27] and consistent with a review of observational studies [27]. Reviews of RCTs are all dominated by the very large trial in Canada [28]. The protocol for this trial specified that women randomised to induction could have prostaglandin gel to ripen the cervix if needed, but women randomised to expectant management who developed an indication for induction could not. This means that women in the expectant arm with an indication for induction and an unfavourable cervix needed CS for delivery. Around one third of women randomised to induction entered labour spontaneously, and one third of those randomised to expectant management needed to be induced. The finding of a reduction in CS with a policy of induction at 41 weeks is therefore not surprising.

Though not as persuasive as well-conducted randomised controlled trials (which are lacking), large population-based datasets provide an opportunity to investigate questions in maternity care with some confidence because their completeness ensures representativeness and minimises bias, unlike voluntary surveys where non-response is seldom random.

A strength of this study is the high level of accuracy of the variables used in the analysis as assessed in a validation study of a random sample of births in 2003. Indication for induction was reported accurately in 95 % of cases and other variables used here were reported accurately in 95–99 % of cases [26].

Analyses of routinely-collected data are limited by the variables available in the dataset. This analysis benefits from the inclusion of the indication for induction of labour in the VPDC. This data set also has the advantage of allowing separate identification of those whose labour was induced and those whose labour was augmented. Although some of the same techniques are used in both cases (amniotomy and oxytocin infusion) the effect when used for induction could differ from augmentation because in the case of induction, the body has not undergone any known or unknown changes that occur physiologically in preparation for labour. The multivariate analysis demonstrated that there were considerably different risks of CS associated with the same techniques according to whether they were used to augment or induce labour.

There are a number of factors that might have been influential but were not available in the VPDC for this analysis including method of fetal monitoring in labour, maternal body mass index, length of labour, preferences of the mother and maternity care providers, time of day and day of the week and model of maternity care.

Some of these items have been added to the perinatal data collection in Victoria at the beginning of 2009, which will enhance future similar analyses.

Induction of labour without medical indication was able to be accurately identified from this detailed and validated dataset, but one limitation of this analysis is that the indication for augmentation of labour was not collected. As a result, we were not able to distinguish a labour that was inco-ordinate, prolonged, or otherwise in undisputed need of augmentation from one that had no such indication for augmentation. For this reason, the results related to induction are more persuasive than those for augmentation.

This analysis compares outcomes for “standard primiparae” who had labour induced with those who did *not* have labour induced (adjusting for important confounders including gestation). This provides evidence of the outcomes of the induction techniques, which is useful information akin to a *per protocol* analysis. Another analysis method could provide a comparison between induction at a particular point in time compared with not inducing at that time (which could result in spontaneous labour on that or a later date, or in induction on a later date). Since gestation was reported only in completed weeks in this dataset, it was not possible to accurately conduct this analysis. Such analysis is planned now that gestation is reported in days.

Like all observational studies, there may well be other unmeasured factors affecting the association between these variables.

The possible mechanism/s for the relationship between induction of labour and CS are beyond the scope of this descriptive paper.

## Conclusion

Using a population dataset with demonstrated accuracy, we found strong evidence of association between elective induction of labour by any of the commonly used methods and birth by CS, which remained significant after adjustment for important confounders. Augmentation of labour that began spontaneously was also associated with an increased risk of CS. Women in this study had no apparent indication for induction of labour, so the increase in CS is not due to underlying risk factors identifiable in the Victorian perinatal data collection. These results suggest that, in the absence of well-designed, contemporary RCTs, minimising unindicated inductions of labour before 41 weeks’ gestation has the potential to reduce the rate of CS without increasing adverse perinatal outcomes.
